# Neural Correlates of Temporal Complexity and Synchrony during Audiovisual Correspondence Detection

**DOI:** 10.1523/ENEURO.0294-17.2018

**Published:** 2018-01-19

**Authors:** Oliver Baumann, Joyce M. G. Vromen, Allen Cheung, Jessica McFadyen, Yudan Ren, Christine C. Guo

**Affiliations:** 1 Queensland Brain Institute, The University of Queensland, St Lucia, Queensland 4072, Australia; 2 QIMR Berghofer Medical Research Institute, Herston, Queensland 4006, Australia; 3 School of Automation, Northwestern Polytechnical University, Xi’an Shi 710065, China

**Keywords:** audiovisual, fMRI, human, multisensory

## Abstract

We often perceive real-life objects as multisensory cues through space and time. A key challenge for audiovisual integration is to match neural signals that not only originate from different sensory modalities but also that typically reach the observer at slightly different times. In humans, complex, unpredictable audiovisual streams lead to higher levels of perceptual coherence than predictable, rhythmic streams. In addition, perceptual coherence for complex signals seems less affected by increased asynchrony between visual and auditory modalities than for simple signals. Here, we used functional magnetic resonance imaging to determine the human neural correlates of audiovisual signals with different levels of temporal complexity and synchrony. Our study demonstrated that greater perceptual asynchrony and lower signal complexity impaired performance in an audiovisual coherence-matching task. Differences in asynchrony and complexity were also underpinned by a partially different set of brain regions. In particular, our results suggest that, while regions in the dorsolateral prefrontal cortex (DLPFC) were modulated by differences in memory load due to stimulus asynchrony, areas traditionally thought to be involved in speech production and recognition, such as the inferior frontal and superior temporal cortex, were modulated by the temporal complexity of the audiovisual signals. Our results, therefore, indicate specific processing roles for different subregions of the fronto-temporal cortex during audiovisual coherence detection.

## Visual Abstract

**Figure:**
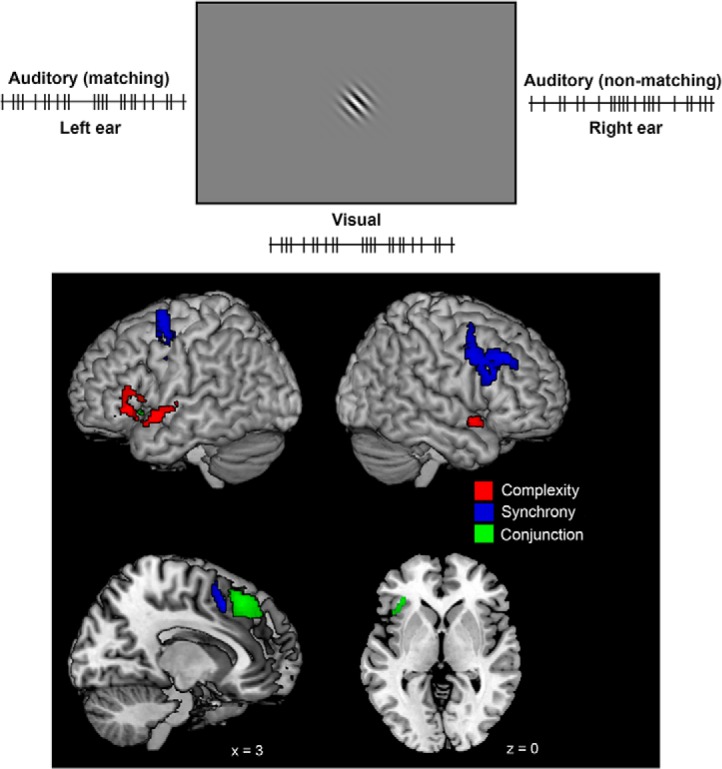

## Significance Statement

The brain’s capability to rapidly integrate signals from different sensory sources lies at the very heart of understanding neural information processing. The temporal structure of sensory events is an important sensory property for the identification of perceptual correspondence. The brain processes underlying their analysis, however, remain unclear. This study shows that modulating the temporal complexity and synchrony of audiovisual streams independently affects neural activity in two distinct sets of brain regions. Our study provides novel neuroanatomical information critical for understanding the mechanisms underlying multisensory integration in the human brain.

## Introduction

Most events in everyday life are perceived simultaneously by using different sensory systems. The sight and sound of a person speaking or a ball bouncing are readily perceived as coherent events but are actually the product of complex neuronal processing. A key task of multisensory processes is to calculate the correspondence between inputs from different senses, thus determining whether two or more perceptual streams are related or not. Previous studies demonstrated that behavioral detection and identification of visual and auditory stimuli are facilitated when they are perceived as coincident in time (Soto-Faraco et al., 2004).

The complexity of audiovisual events ranges from simple flash-beep stimuli to the integration of speech and gesture. A number of recent studies have suggested that the perception of audiovisual correspondence is modulated by the complexity of the temporal profile of perceptual events. For instance, more complex audiovisual stimuli such as natural speech allow for larger temporal discrepancies (i.e., a larger window of integration of up to ∼260 ms) than simple stimuli (i.e., ∼60- to 70-ms window of integration; compare Vatakis and Spence, 2010). In a dedicated study to investigate this phenomenon, Denison et al. (2013) used rapid streams of auditory and visual events to demonstrate that stochastic, irregular streams, i.e., with richer temporal pattern information, did in fact lead to higher audiovisual matching sensitivity than predictable, regular streams. That is, the observers’ matching performance benefitted from the higher information content provided by the streams with more complex temporal structure. Interestingly, the contributions of temporal asynchrony and complexity to perceptual sensitivity were statistically independent (Denison et al., 2013). It remains unknown, however, whether the cognitive demands placed by increments in temporal asynchrony and decrements in temporal complexity are supported by a common or diverse set of brain regions. The discovery of separate neural correlates for processing temporal complexity and synchrony of stimulus streams would provide evidence against the assumption of an unspecific task difficulty effect.

**Figure 1.**
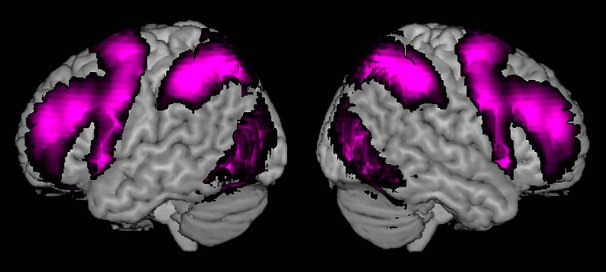
Neuroanatomical extend of the multiple demand network or MD cortex, based on averaged unthresholded activity of seven diverse task sets (Fedorenko et al., 2013).

It had been proposed that a common neural system, the so-called cognitive control network (CCN, Cole and Schneider, 2007), also referred to as the frontoparietal control system (Vincent et al., 2008) or the multiple demand system (Duncan, 2010), underlies performance in many cognitive tasks. The core CCN system is thought to extend over a specific set of regions in prefrontal and parietal cortex, including the dorsolateral prefrontal cortex (DLPFC) inferior frontal sulcus (IFS), the anterior insula and adjacent frontal operculum (AI/FO), as well as the pre-supplementary motor area and adjacent dorsal anterior cingulate cortex (pre-SMA/ACC), and the intraparietal sulcus (IPS). Figure 1 displays the neuroanatomical extent of the network based on averaged activity of seven diverse task sets (Fedorenko et al., 2013). The components of the CCN system are commonly activated together, but the differential structural connectivity profile of the involved regions with other parts of the brain could be suggestive of region-specific processing roles, an idea that is further corroborated by a number of functional imaging studies. For instance, it has been shown that DLPFC responds to working memory demands (Barch et al., 1997; Manoach et al., 1997; Smith et al., 1998) while it has been proposed that the IPS is of particular importance for multisensory integration (Cusack 2005) as well as spatial attentional processes (Coull et al., 2000; Materna et al., 2008).

In this study, we aimed to identify differential activity in subregions of the CCN with regard to variations in perceptual asynchrony and complexity of audiovisual stimulus streams. Furthermore, perceptual coherence matching requires highly precise temporal processing. Several lines of evidence suggest that the cerebellum is an essential component in processing precise temporal relationships in the subsecond range for sensorimotor as well as perceptual tasks (Baumann et al., 2015). Thus, we hypothesised that activity in the cerebellum would be modulated by variations in complexity as well as synchrony. Finally, on a more pragmatic level, our study intended to identify the neural correlates underlying the decrement in task performance associated with high degrees of perceptual asynchrony and low degrees of complexity that were identified in the study by Denison et al. (2013). By identifying brain structures that are differentially activated by variations in stimulus complexity and synchrony, our study sought to provide novel information critical for understanding the functional neuroanatomy underlying audiovisual coherence detection.

## Materials and Methods

First, we aimed to replicate the effects of the multisensory stimulus design of Denison et al. (2013) in a psychophysical pilot experiment (24 participants) and then assessed the neural responses in the fMRI scanner (34 participants). Finally, we conducted a psychophysical control experiment (24 participants) to address a potential challenge to the results.

### Psychophysical experiment

#### Participants

Twenty-four healthy volunteers (18 female), ranging in age from 17 to 24 (mean 19 years), gave their informed consent to participate in the experiment, which was approved by the Human Research Ethics Committee of the University of Queensland.

#### Stimuli and apparatus

**Figure 2.**
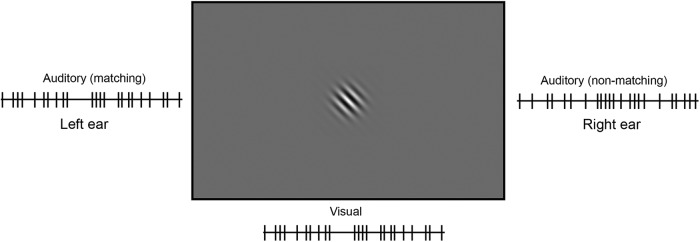
Schematic representation of the audiovisual matching task. Participants listened to two different auditory stimulus streams (rapid pips), one presented to each ear, while simultaneously viewing a centrally presented visual stimulus stream (left-right flipping Gabor grating). One of the auditory streams matched the temporal profile of the visual stream and participants had to indicate its source (left or right ear).

We modified the approach by Denison et al. (2013) into a design suitable for fMRI. Denison et al. (2013) employed two simultaneous visual streams, which could entice participants to break fixation, especially under more difficult task conditions. This in turn could lead to potentially confounding eye movement related activity differences when using fMRI. To minimize potential eye movement confounds, we employed two simultaneous auditory streams (left and right ear) and just one centrally presented visual stream (Fig. 2).

The visual stimulus was a centrally presented Gabor grating (truncated squarely at 6.9 × 6.9 cm) on a medium gray background. Each grating was created by multiplying a circular Gaussian mask (FWHM 2.05 cm) to a 100% contrast black and white oriented sine-wave grating with spatial frequency of 1 cycle per 0.82 cm. The grating subtended 6.58° of visual angle (1 cycle 0.78° of visual angle). Visual stimulus streams were generated by presenting orthogonal oriented gratings (+45/−45°) in alternation, i.e., resulting in a tilted grating flipping back and forth in orientation over time. Auditory stimuli were sequences of 10-ms tone pips with frequencies of 500- and 1000-Hz pure tones (one presented to the left ear and one to the right ear, respectively). The tones had technically imposed on/off ramps of 5 ms.

The psychophysical experiment was conducted in a sound attenuated chamber and stimuli were presented using the Psychophysics Toolbox for MATLAB (Mathworks Inc.). Visual stimuli were presented on an ASUS LCD monitor (refresh rate 60 Hz, resolution 1920 × 1080, screen size 53 × 30 cm) at a 60-cm viewing distance. Auditory stimuli were presented using Noontec Zero headphones (Noontec, Australia) at a comfortable listening volume.

#### Generation of stimulus streams by a stochastic point process

For each trial, one visual stream and two auditory streams were generated using a stochastic point process. Each trial was divided into 40 equally spaced time bins of 100-ms duration. At the start of each time bin, an event (tone pip or flip of Gabor filter) occurred with a probability of 1/3, resulting in streams of events separated by variable interstimulus intervals (ISIs). In every trial, one of the auditory streams matched the visual stream, i.e., the two streams had identical temporal event profiles. The nonmatching auditory stream was generated by shuffling the ISI of the matching stream, thereby generating a new temporal pattern but with identical ISI frequency distribution.

#### Complexity manipulation

Following Denison et al. (2013), we controlled three types of complexity using information theoretic measures: (1) first-order event entropy (the presence or absence of an event at a given time point); (2) first-order ISI entropy (ISI variability); and (3) second-order ISI entropy (ISI sequence variability). First-order event entropy was kept constant in our experiment, while second-order and third-order entropy were systematically varied.

First-order event entropy reflects information contained in the presence and absence of sensory events at any given time point. This type of entropy therefore represents uncertainty about the occurrence of an event. The entropy for a time bin *X* is calculated according to Equation 1, where *x_i_*is the event state (0 or 1), and *p*(*x_i_*) is the probability of *x_i_* occurring, given the event distribution of the stimulus stream. Since all streams in our experiment were generated with a fixed probability for events per time bin of 1/3, the theoretical first-order entropy is 0.92 bits (per time bin) for an infinite sequence, and was 0.93 bits on average over our sequences.

(1) $$H\left(X\right)=-\overset{n}{\underset{i=1}{\sum }}p\left(x_{i}\right){log}_{2}\left(p\left(x_{i}\right)\right)$$

First-order ISI entropy reflects the complexity of a stream arising from the variability of the temporal intervals within a stimulus stream. When applying Equation 1 to calculate second-order entropy, *x_i_* denotes each possible ISI (time between successive event onsets). Streams with more variable ISIs will have high values of entropy (i.e., complexity) and provide a higher degree of uncertainty regarding the duration of any given ISI.

Second-order ISI entropy reflects the complexity provided by the sequential structure of stimulus streams. Applying Equation 1, *x_i_* now denotes each unique successive ISI pair in stimulus streams. For example, a stream with only two unique ISI presented in a fixed alternating order would have lower third-order entropy than a stream in which two unique ISI are presented in a random order.

For our high-complexity condition, we generated stochastic stimulus streams as described above, with a fixed event probability of 1/3. Nonmatching streams were generated by randomly shuffling the matching streams, thereby producing different temporal patterns but matched for ISI variability. For our low-complexity condition, we first generated a so-called “bar” comprising five ISIs using the same stochastic process as before. This bar was then replicated and strung together to again generate streams with 40 bins. To create nonmatching streams, a new rhythm based on a shuffled version of the bar was used. The nonmatching bar comprised the same five ISIs as for the original bar but constrained to be in a different order.

Using this method, we discovered that low- and high-complexity streams not only differed in their entropy, but also in the amount of mutual information shared between matching and nonmatching streams of each condition.

Mutual information *I* between two streams *X* and *Y* was calculated according to Equation 2, where *H*(*X*) is the entropy of X (Eq. 1) and H(X|Y) is the conditional entropy of X given Y. Conditional entropy is calculated according to Equation 3, where X and Y are simultaneous bins from two streams presented in the same trial.

(2) $$I\left(X;Y\right)=H\left(X\right)-H\left(X|Y\right)$$

(3) $$H\left(X|Y\right)=\overset{n}{\underset{i=1}{\sum }}\overset{m}{\underset{j=1}{\sum }}p\left(x_{i},y_{j}\right){log}_{2}\left(\frac{p\left(y_{i}\right)}{p\left(x_{i},y_{j}\right)}\right)$$

**Figure 3.**
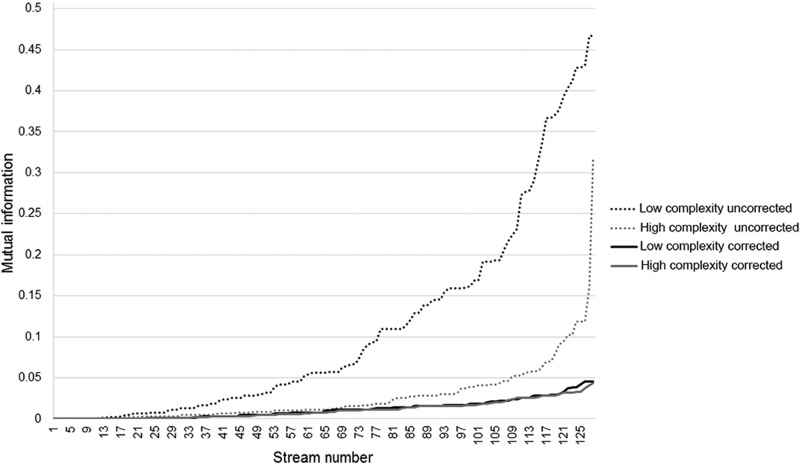
Distribution of mutual information of the high and low complexity stimulus streams before and after correction (installing a maximum limit of 0.05 bits).

**Figure 4.**
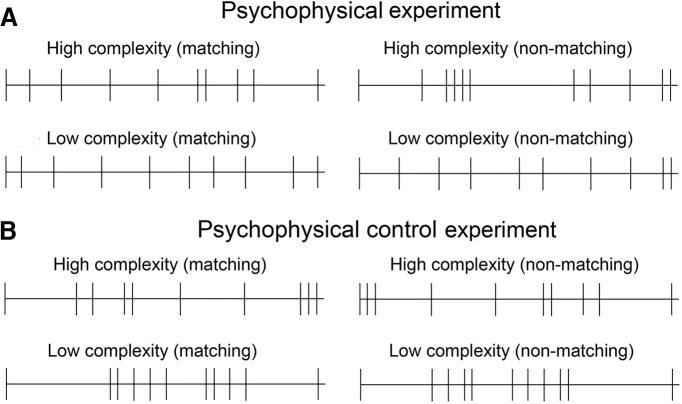
Visual representations of typical examples of the 4-s temporal sequences used for the two levels of complexity in (***A***) the psychophysical experiment and (***B***) the psychophysical control experiment.

Specifically, the low-complexity condition had consistently higher amounts of mutual information than the high-complexity condition (Fig. 3). In other words, the matching and nonmatching stream in the low-complexity condition were more similar than in the high-complexity condition, which could differentially affect the task difficulty for both conditions. To control for this potential confound, we generated the streams while applying a maximal limit of 0.05 bits of mutual information between streams, i.e., any stream exceeding this value was discarded and replaced. Using this approach, we were able to create high- and low-complexity streams that did not differ in mutual information (Fig. 3). The resulting high complexity stimulus streams had an average first-order ISI entropy of 2.18 bits, and an average second-order ISI entropy of 3.21 bits. In contrast, the low-complexity streams had an average first-order ISI entropy of 1.80 bits, and second-order ISI entropy of 2.42 bits (for examples of the temporal sequences used for the two levels of complexity in the psychophysical experiment, see Fig. 4*A*).

#### Synchrony manipulation

To manipulate the synchrony of the visual and auditory streams, the auditory streams were shifted relative to the visual streams in equal proportions by 0, +100, +200, and +300 ms. Therefore, the auditory streams lagged behind the visual stream in 75% of the trials. Asynchronous matching events that extended beyond the fixed stream duration due to the lag were looped back to the corresponding positions in the beginning of the stream, preventing long gaps in stimulation at the start of the trial. In addition, the first and last time bins of each trial always contained an event in all three streams for all conditions, thereby preventing participants from simply orienting to initial or terminal events.

#### Task

Participants listened to two streams (500 and 1000 Hz) of auditory events (4-s sequences of tone pips). One stream was presented to the left ear and the other to the right ear. For half of the participants, 500-Hz pips were presented to the left and 1000-Hz pips to the right ear and vice versa. Simultaneously, participants viewed a single stream of visual events, i.e., a 45° tilted Gabor grating flipping back and forth by 90°. On each trial, the temporal pattern of orientation changes of the Gabor grating matched the temporal pattern of one of the auditory streams (i.e., the matching stream), but not the other (i.e., the nonmatching stream). As described earlier, the matching and nonmatching streams were generated using the same stochastic process and were equivalent concerning their temporal statistics. Using a two-alternative forced-choice task, participants were asked to indicate via button press within two seconds after the end of the stimulation which of the two auditory streams (left or right ear) matched the temporal pattern of the visual stream. Following the response period, participants were given feedback via a 1-s screen message (i.e., either “hit,” “miss,” or “too slow”), before the next trial started.

The psychophysical experiment was divided into eight ∼4-min blocks that contained 32 trials each, i.e., four trials per condition, as defined by complexity (high or low), and synchrony (0, +100, +200, or +300 ms). In total, there were 256 trials, resulting in 32 per unique condition presented in randomized order. Before the start of the experiment, participants completed two practice blocks to familiarize them with the task.

#### Statistical analysis

The behavioral data were analyzed using repeated two-way repeated-measures ANOVA implemented via IBM SPSS 22 (for details on the statistical tests, see Table 1).

**Table 1.** Results of the two-way repeated-measures ANOVA for the psychophysical experiment

Factor	*F*(*df*)	*p* value	**partial-η*^*2*^*
Main effect: synchrony	25.37 (3,72)	<0.001	0.514
Main effect: complexity	40.06 (1,24)	<0.001	0.626
Interaction: synchrony × complexity	3.96 (3,72)	0.011	0.142

### fMRI experiment

#### Participants

Thirty-four healthy volunteers (16 female), ranging in age from 18 to 27 (mean 21 years), gave their informed consent to participate in the experiment, which was approved by the Human Research Ethics Committee of the authors’ institution.

#### Stimuli and procedure

The stimuli were identical to the psychophysical experiment with the exception that visual stimuli were presented at a greater distance (i.e., 90 cm). Thus, the Gabor stimuli subtended 4.39° of visual angle (1 cycle per 0.52° of visual angle). Stimuli were presented using Cogent toolbox under MATLAB (Mathworks Inc.) and a liquid crystal display projector (60-Hz refresh rate, resolution 1920 × 1080 pixels) that back-projected stimuli onto a screen positioned at the head end of the scanner bed. Participants lay on their back within the bore of the magnet and viewed the stimuli via a mirror that reflected the images displayed on the screen. The distance to the screen was 90 cm (12 cm from eyes to mirror) and the visible part of the screen encompassed ∼22.0 × 16.4° of visual angle (35.5 × 26 cm). The auditory stimuli were presented at a comfortable listening volume using a MR Confon MR-compatible sound system (MR Confon, GmbH).

#### Complexity and synchrony manipulation

As in the behavioral version of the task, there were two levels of complexity (high vs low). To increase trial numbers for each condition, we employed two levels of synchrony (i.e., 0 vs +200 ms) instead of the four in the psychophysical experiment.

#### Task

The task was mostly identical to the psychophysical experiment. However, we increased the ISI from three to four seconds which enabled a central fixation cross to be displayed for 1 s after the feedback period.

The experiment was divided into seven ∼6-min blocks that contained 40 trials each, i.e., 10 trials per condition, as defined by complexity (high or low) and synchrony (0 or 200 ms). In total, there were 280 trials, resulting in 70 per unique condition presented in random order. Before the start of the experiment, participants completed one practice block while lying in the scanner to familiarize them with the scanner environment.

#### MRI acquisition

Brain images were acquired on a 3T MR scanner (Trio; Siemens) fitted with a 12-channel head coil. For the functional data, 44 axial slices (slice thickness, 3 mm; gap 10%) were acquired in a descending order using a gradient echo echoplanar T2*-sensitive sequence (repetition time, 2.68 s; echo time, 30 ms; flip angle, 80°; matrix, 64 × 64; field of view, 192 × 192 mm; in-plane resolution, 3 × 3 mm; phase encoding polarity, positive). In each of seven runs, 126 volumes were acquired for each participant; the first two images were discarded to allow for T1 equilibration. Geometric distortions in the EPI images caused by magnetic field inhomogeneities were corrected using a point-spread mapping approach (Zeng and Constable, 2002; Zaitsev et al., 2003). We also acquired a T1-weighted structural MPRAGE scan. To minimize head movement, all participants were stabilized with tightly packed foam padding surrounding the head.

#### Data analysis

The behavioral data were analyzed using two-way repeated-measures ANOVA implemented via IBM SPSS 22 (for details on the statistical tests, see Table 2). Image processing and statistical analyses were performed using SPM12 (Wellcome Department of Imaging Neuroscience, University College London). Functional data volumes were slice-time corrected and realigned to the first volume. A T2*-weighted mean image of the unsmoothed images was coregistered with the corresponding anatomic T1-weighted image from the same individual. The individual T1 image was used to derive the transformation parameters for the stereotaxic space using the SPM12 template (Montreal Neurologic Institute template), which was then applied to the individual coregistered EPI images. In the first-level analysis, we generated a model incorporating four experimental task regressors: (1) low complexity, 0-ms delay; (2) low complexity, 200-ms delay; (3) high complexity, 0-ms delay; and (4) high complexity, 200-ms delay. In the second-level analysis, to identify condition-specific difficulty effects associated with the two task factors, we employed a combination of paired *t* tests (height-threshold *p* = 0.0001, extent-threshold *p* = 0.05, FWE-corrected for multiple comparisons) and exclusive masking (masking threshold *p* = 0.05, uncorrected). Specifically, we computed the contrast “low complexity > high complexity” (combining delays of 0 and 200 ms) and masked it with the contrast “200 > 0 ms” (combined for low complexity and high complexity), and vice versa. It is important to note that a liberal mask for an exclusive mask is more conservative in excluding common regions from the statistical parametric map. Second, to identify potential interactions between the task factors, we conducted a SPM full factorial ANOVA with factors of complexity (high, low) and synchrony (0, 200 ms). Finally, to identify common activity related to task difficulty, we conducted a conjunction analysis (using a conjunction-null hypothesis) across these task conditions relative to the implicit baseline (Nichols et al., 2005; height-threshold *p* = 0.0001, extent-threshold *p* = 0.05, FWE corrected). The anatomic locations of activity clusters were determined using the xjView toolbox.

**Table 2.** Results from two-way repeated-measures ANOVA for the fMRI experiment

Factor	*F*(*df*)	*p* value	**partial-η*^*2*^*
Main effect: synchrony	47.15 (1,33)	<0.001	0.589
Main effect: complexity	448.89 (1,33)	<0.001	0.932
Interaction: synchrony × complexity	3.59 (1,33)	0.067	0.098

### Psychophysical control experiment

#### Participants

Thirty-four healthy volunteers (17 female), ranging in age from 18 to 33 (mean 22 years), gave their informed consent to participate in the experiment, which was approved by the Human Research Ethics Committee of the authors’ institution.

#### Stimuli and procedure

The concept of complexity is synonymous with disorderliness, which means that there is necessarily more repetition in low-complexity sequences than high-complexity sequences. However, it could be argued that decreased accuracy in the low-complexity condition is driven by the strict rhythmicity due to the use of repeating bars, rather than low entropy per se. To investigate this potential challenge to our results, we conducted this control experiment, in which the stimuli and procedure were identical to the first psychophysical experiment, with the exception that the stimulus sequences in the low-complexity condition were generated without repeating bars using a three-step procedure. First, we generated 5000 independent stochastic streams, again with an event probability of 1/3 and 40-time bins of 100 ms. Second, we summed the first-order and second-order ISI entropies of each stream and its nonmatching counterpart and extracted the top and bottom deciles. Finally, we computed the event entropy of each stream from the upper and lower deciles identified streams from the lower decile that had an exact event entropy match in the upper decile group. The resulting high complexity stimulus streams had an average first-order ISI entropy of 2.44 bits, and an average second-order ISI entropy of 3.26 bits. In contrast, the low-complexity streams had an average first-order ISI entropy of 1.94 bits, and second-order ISI entropy of 2.81 bits (for examples of the temporal sequences used for the two levels of complexity in the psychophysical control experiment, see Fig. 4*B*).

#### Statistical analysis

The behavioral data were analyzed using repeated two-way repeated-measures ANOVA implemented via IBM SPSS 22. Please see Table 3 for details on the statistical tests conducted.

**Table 3.** Results from two-way repeated-measures ANOVA for the psychophysical control experiment

Factor	*F*(*df*)	*p* value	**partial-η*^*2*^*
Main effect: synchrony	85.76 (3,72)	<0.001	0.789
Main effect: complexity	17.76 (1,24)	<0.001	0.436
Interaction: synchrony × complexity	5.14 (3,72)	0.003	0.183

## Results

### Psychophysical experiment

**Figure 5.**
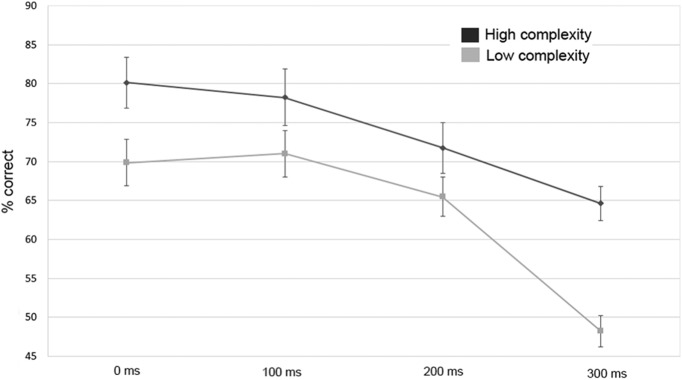
Accuracy (±1 SEM) for matching performance in the psychophysical experiment, separately for each level of the complexity (dark = for high complexity, light = low complexity) and asynchrony manipulations (0, 100, 200, and 300 ms offsets).

The outcomes from this experiment (Fig. 5) broadly replicated the results reported by Denison et al. (2013). A repeated-measures ANOVA showed that audiovisual correspondence detection accuracy declined with increasing degrees of asynchrony (*F*_(3,72)_ = 25.37 *p* < 0.001, *partial-η^2^* = 0.514). Further, accuracy was significantly higher in the high-complexity condition compared to the low-complexity condition (*F*_(1,24)_ = 40.06, *p* < 0.001; *partial-η^2^* = 0.626). In contrast to Denison et al. (2013), we also observed a significant medium-sized interaction between the effects of complexity and synchrony (*F*_(3,72)_ = 3.96, *p* = 0.011, *partial-η^2^* = 0.142). Figure 5 shows that the interaction was driven by the fact that the difference between the high- and low-complexity conditions was particularly large at the highest level of asynchrony (i.e., accuracy for the low-complexity condition was 48.25%, at chance, for the streams delayed by 300 ms). In summary, the psychophysical data show that our design (i.e., using two auditory streams and one visual stream) lead to equivalent performance with Denison et al. (2013)’s design (i.e., using two visual and one auditory stream). This suggests that the effects of synchrony and complexity on audiovisual coherence detection are both reliable and robust to differences in experimental paradigm.

### fMRI experiment

**Figure 6.**
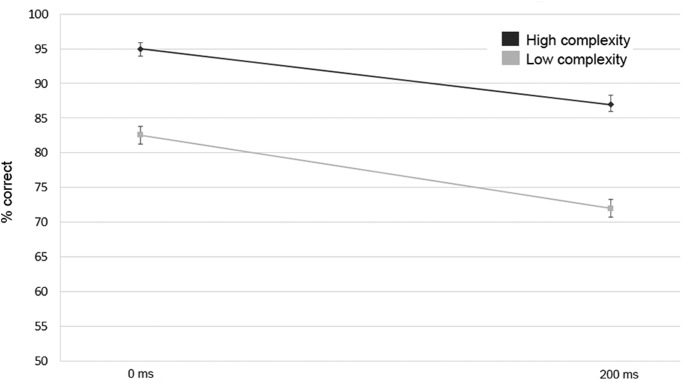
Accuracy (±1 SEM) for matching performance in the fMRI experiment, separately for each level of the complexity and synchrony manipulations.

The detection accuracy in the fMRI experiment (Fig. 6) followed an overall similar pattern but was notably better than in the psychophysical experiment. This could be either a group effect (i.e., a different set of participants was employed) or the absence of the very difficult 300-ms delay condition led to decreased task uncertainty and higher overall task proficiency.

Coherence detection performance was higher for synchronous streams (0-ms delay) compared to asynchronous (200-ms delay) streams (*F*_(1,33)_ = 47.15, *p* < 0.001, *partial-η^2^* = 0.589), as well as for high-complexity compared to low-complexity streams (*F*_(1,33)_ = 448.89, *p* < 0.001, *partial-η^2^* = 0.932). There was, however, no statistically-significant interaction between the two task factors (*F*_(1,33)_ = 3.59, *p* = 0.067, *partial-η^2^* = 0.098).

For the fMRI experiment, we also acquired response time data and found that participants responded faster in the synchronous condition (*F*_(1,33)_ = 39.36, *p* < 0.001, *partial-η^2^* = 0.54), but there was no significant difference in complexity (*F*_(1,33)_ = 1.10, *p* = 0.30, *partial-η^2^ = 0.032*), nor an interaction between the two task factors (*F*_(1,33)_ = 0.32, *p* = 0.576, *partial-η^2^ = 0.010*).

**Figure 7.**
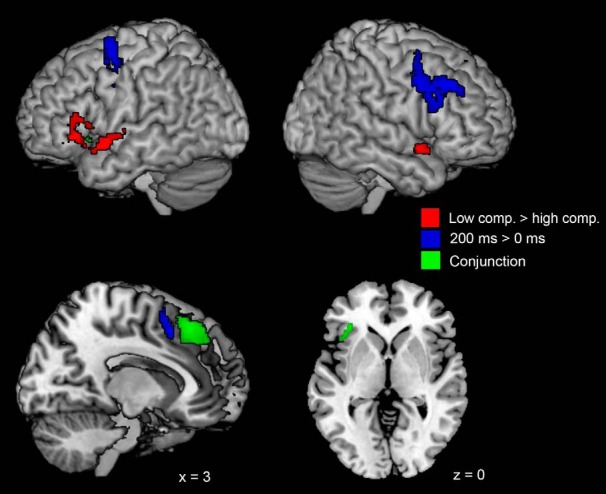
Results of the fMRI random effects analysis showing unique activation patterns for the complexity manipulation (low complexity > high complexity, shown in red), synchrony manipulation (200-ms delay > 0-ms delay, shown in blue) using an exclusive masking approach. Common activity for both manipulations (i.e., via conjunction analysis) is shown in green.

The fMRI analysis revealed condition-specific activation patterns (Fig. 7; Table 4). The high-complexity condition yielded greater activity in the left inferior frontal gyrus (i.e., Broca’s area) and in the bilateral superior temporal gyri. In contrast, the performance effects due to asynchrony in the audiovisual streams were associated with increased activity in the left DPFC, the SMA, and the bilateral precentral gyri.

**Table 4.** Summary of fMRI findings for three contrasts of interest

Region	Hemisphere	Brodmann area	MNI coordinates			T values/z values of maxima (cluster size in number of voxels)
			X	Y	Z	
Low complexity > high complexity (masked exclusively by 200 > 0 ms)						
IFG (tri/orb)	L	45/47	−40	30	−3	5.70/5.38 (211)
STG	L	22/38	−50	4	−7	5.53/5.23 (159)
STG/TP	R	38	50	8	−11	5.24/4.98 (79)
200 > 0 ms (masked exclusively by high complexity > low complexity)						
Pre-SMA/MedFG/SFG/Cing	L + R	6/32	6	14	47	5.93/5.57 (136)
MFG/PrecG	L	6	−30	−2	55	5.70/5.38 (452)
MFG/PrecG/IFG (Oper/Tri)	R	9/8/6	46	32	37	5.55/5.25 (929)
Conjunction: (low complexity > high complexity) Λ (200 > 0 ms)						
Pre-SMA/SFG(medial)	L + R	8/6/32	2	28	47	5.57/5.27 (599)
IPL	L	40/7	−30	−50	45	5.01/4.78 (160)
Ins/IFG(tri/orb)	L	47/45	−34	30	1	4.95/4.73 (91)

Spatial coordinates, anatomic locations, and cluster size of the local maxima in the group analysis, showing significant activations (height-threshold *p* = 0.0001, extent-threshold *p* = 0.05, FWE-corrected for multiple comparisons). CinG = cingulate gyrus, IFG = inferior frontal gyrus, Ins = insula, IPL = inferior parietal lobule, L = left hemisphere, MedFG = medial frontal gyrus, MFG = middle frontal gyrus, Oper = opercularis, PrecG = precentral gyrus, R = right hemisphere, SFG = superior frontal gyrus, STG = superior temporal gyrus, TP = temporal pole, tri = triangularis.

In addition to condition-specific patterns, the analysis also identified three activation clusters that were common to both the low complexity as well as the asynchronous conditions. These were located in the left inferior parietal cortex, the left insula, as well as medially in the SMA (Fig. 7, green). Finally, the factorial analysis (which aimed at identifying potential interactions between the two task factors) did not yield any significant results.

### Psychophysical control experiment

**Figure 8.**
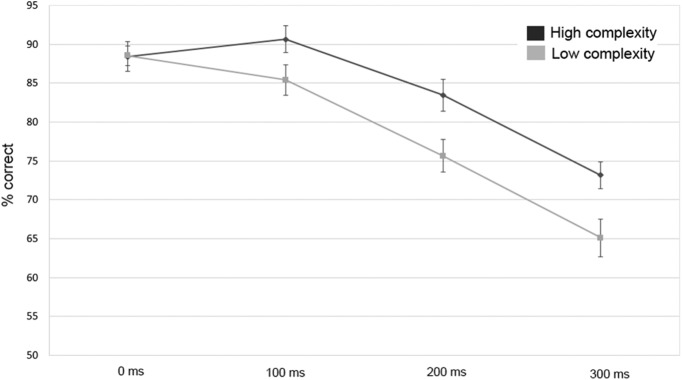
Accuracy (±1 SEM) for matching performance in the psychophysical control experiment, separately for each level of the complexity and asynchrony manipulations.

The outcomes from the control experiment (Fig. 8) broadly replicated the results from our other two experiments. A repeated-measures ANOVA showed that audiovisual correspondence detection accuracy declined with increasing degrees of asynchrony (*F*_(3,72)_ = 85.77 *p* < 0.001, *partial-η^2^* = 0.789). Further, accuracy was significantly higher in the high-complexity condition compared to the low-complexity condition (*F*_(1,24)_ = 17.76, *p* < 0.001; *partial-η^2^* = 0.436). As in the first psychophysical experiment, we observed a significant interaction between the effects of complexity and synchrony (*F*_(3,72)_ = 5.14, *p* = 0.003, *partial-η^2^* = 0.183). Figure 8 shows that the interaction was driven by the fact that the difference between the high- and low-complexity conditions was only evident for the asynchronous conditions. The absence of a complexity effect for the synchronous conditions could potentially be caused by a ceiling effect (i.e., ∼88% in both conditions), given the overall higher level of performance compared to the first psychophysical experiment (i.e., 80.1% in the synchronous high-complexity condition). We observed overall higher levels of performance in the fMRI experiment, but given the lower number of synchrony levels in the fMRI experiment, performance might not be directly comparable. In summary, the control experiment shows that the significantly reduced performance in the low-complexity condition cannot be due to repeating bars, since there were no repeating bars in this control experiment.

## Discussion

Our study confirmed the findings of Denison et al. (2013), showing that participants’ ability to match the temporal structure of audiovisual streams is modulated by the complexity of the signals as well as their degree of synchrony. Specifically, increased levels of entropy lead to better matching performance while increased levels of asynchrony lead to worse performance. In contrast to Denison et al. (2013), we observed an interaction between complexity and synchrony. The interaction, however, was small in comparison to the robust main effects.

While the fact that asynchrony leads to performance decrements is intuitively appealing, this is not the case for the inverse relationship between signal complexity and accuracy. The comparison of multiple perceptual streams is expected to involve short-term memory processes, which should be facilitated by lower signal complexity, i.e., due to decreased memory load. The pattern of results can be explained, however, from an information theory perspective, supposing that observers would benefit from the higher information content of the high-complexity streams because two complex streams are less likely to have the same temporal pattern by chance than a simple stream.

Our fMRI experiment aimed to identify the neural correlates underpinning impaired matching ability associated with high degrees of perceptual asynchrony and low degrees of complexity. Of particular interest was whether, and to what degree, the two task factors were underpinned by common brain regions. Our conjunction analysis revealed common difficulty-related activity in the SMA, the inferior parietal lobule, and the AI. As mentioned earlier, all these three regions are part of the so-called multiple-demand CCN (Cole and Schneider, 2007). In contrast, the condition-specific analysis revealed that brain activity patterns related to increased task difficulty differed for the factors of complexity and synchrony. Complexity related activity was located mainly in pars triangularis and orbitalis of the left inferior frontal gyrus, as well as the left superior temporal gyrus. In contrast, synchrony-related activations were located primarily in the right DLPFC and the bilateral precentral gyri.

The activation patterns associated with the complexity modulation span brain regions known to underpin multisensory integration as well as speech recognition and production. The superior temporal gyrus is known as the site of the auditory association cortex and could be activated by audiovisual coherence detection tasks (Baumann and Greenlee, 2007) as well as the processing of word meaning (Booth et al., 2002). Pars triangularis and orbitalis of the left inferior frontal gyrus are commonly known as Broca’s area, a region strongly implicated in various language tasks as demonstrated in neuroimaging and lesion-based studies (for an overview, see Fadiga et al., 2009). Interestingly, however, both Broca’s area as well as the superior temporal gyrus have also been suggested to be involved in the processing of musical structure (Koelsch, 2006). More specifically, a PET study of same-different discrimination of pairs of auditory rhythmic patterns activated Broca’s area, when compared to timbre and pitch data (Platel et al., 1997), suggesting that the analysis of temporal structures is a central function. Furthermore, it had previously been shown that Broca’s area is sensitive to the coherence of audiovisual speech patterns (Ojanen et al., 2005). It is conceivable that this brain region is also involved in the matching of nonspeech rhythmic patterns. It is plausible that the higher effort required for matching low-complexity rhythmic patterns leads to higher neural activity in this area. Alternatively, the lower accuracy rates for low-complexity stimuli could indicate that these stimuli are less likely to be perceived as coherent audiovisual objects, but rather as distinct streams of activation, leading to activation of disparate neuron populations.

The synchrony modulation led to activation in the DLPFC (i.e., Brodmann area 9). Dorsolateral prefrontal cortex is commonly thought to maintain task relevant sensory information in working memory (Goldman-Rakic, 1987; Levy and Goldman-Rakic, 2000). In that context, it is conceivable that asynchrony between the auditory and visual streams leads to an increase in working memory load, since larger proportions of the stimulus streams have to be maintained to allow successful matching. In fact, the asynchronous matching task even shares some characteristics with the popular n-back task. During the task, the streams are delayed by two bins of 100 ms each, so it could be described as a fast-paced and short-term version of a nonverbal cross-modal 2-back task. Increased memory load for nonverbal n-back tasks consistently leads to heightened activity in both the dorsolateral prefrontal cortex and premotor cortex (for an overview, see Owen et al., 2005). It is, however, important to note that our task might engage working memory in a different way due to its rapidity. Notably, increased memory load in more static memory tasks such as the Sternberg paradigm also leads to increased activity in both the dorsolateral prefrontal cortex and the premotor cortex (Altamura et al., 2007). Taken together, previous studies and our current findings suggest that audiovisual asynchrony may have increased DLPFC activation due to increased working memory load.

Furthermore, the conjunction analysis revealed difficulty-related activity increases in the left inferior parietal cortex, the left insula, and in the SMA that were common to both the complexity as well as the synchrony manipulations. The AI has been described as central to the ventral attention system for coordinating task performance (Dosenbach et al., 2007) and is also described as integral hub in mediating dynamic interactions between other large-scale brain networks involved in externally oriented attention and internally oriented or self-related cognition (Menon and Uddin, 2010). On the other hand, lesions of the inferior parietal lobule have been found to lead to deficits specifically in temporal attention, and is thought to provide a top-down control role for nonspatial perception (Shapiro et al., 2002). Furthermore, the SMA is considered critical for performance- and error-monitoring, a process particularly relevant during more difficult task conditions. For example, microstimulation of the SMA during a go or no-go task reduced the frequency of incorrect fast responses and increased the frequency of slower correct ones (Isoda and Hikosaka, 2007).

Previous fMRI studies on audiovisual correspondence matching have focused on identifying the neural correlates related to matching versus nonmatching pairs of perceptual streams (Baumann and Greenlee, 2007; Alink et al., 2008). The study by Baumann and Greenlee (2007) showed that coherent, compared to incoherent, audiovisual streams led to increased activity in the prefrontal, parietal and superior temporal brain cortices. In contrast, we designed the present study to distinguish brain activation patterns related to varying levels of stimulus complexity and synchrony during audiovisual perception. While the activation patterns of our current study and the earlier work by Baumann and Greenlee (2007) overlap, it is not prudent to make cross-experiment comparisons. Future studies should explore how neural correlates of perceptual complexity and synchrony are modulated by different levels of stimulus coherence.

It is also noteworthy that our task did not modulate activity in the cerebellum, a region thought to be essential for temporal processing. Initial evidence for a cerebellar role in timing was provided by Ivry and Keele (1989), who showed that patients with cerebellar pathology were impaired in judging the duration of an auditory stimulus but showed normal performance in judging stimulus loudness. Since then, a number of lesion and imaging studies have suggested that the cerebellum is responsible for representing temporal relationships in the sub-second range for sensorimotor as well as perceptual tasks (Baumann et al., 2015). Apart from a hypothesized role of the cerebellum in temporal processing, there are reports of cerebellar activity during tasks involving audiovisual matching (Baumann and Greenlee, 2007; Bushara et al., 2001; Calvert, 2001). For instance, it has been shown that combined audiovisual motion detection leads to increased activity bilaterally in the cerebellar hemispheres relative to unimodal visual and auditory motion tasks (Baumann and Greenlee, 2007). Therefore, the absence of differential activity in our current study suggests that the cerebellum may have been generally active during the task but was simply not modulated by the changes in signal complexity or synchrony. That is, the different conditions might have led to activation of slightly disparate neuron populations (i.e., dedicated to process signals with different levels of complexity or latency) but not to activation of proportionally larger and more energy intensive neural networks.

Our control experiment showed that the significantly reduced performance in the low-complexity condition was not due to the use of repeating bars, since there were no repeating bars in this control experiment. This further corroborates the assumption that the effect of stimulus complexity on audiovisual coherence detection is reliable and robust to differences in sensory stimulus details. It is further important to note that the concept of complexity is synonymous with disorderliness, which means that low-complexity sequences are necessarily more orderly sequences. In the context of ISI entropies, that means less variability in individual ISIs and consecutive ISI pairs. This, in turn, equates to necessarily more repetition in low-complexity sequences irrespective of whether repeating ISI bars are used.

It is also important to note that our randomized 2 × 2 factor design should make the use of different strategies associated with the complexity and synchrony very unlikely. The two task factors are not tested in isolation (e.g., 50% of the trials have a common (either high or low) demand on synchrony as well as complexity and are presented in a random order, which does not lend itself to the employment of separate strategies for the two factors. Moreover, an earlier study by Maddox et al. (2015) using similar stimuli showed that coherence between auditory and visual perceptual streams can affect performance even if the visual stream is not task relevant, suggesting that audiovisual coherence detection does not require cognitive exertion.

In summary, our study demonstrated that decrements in audiovisual coherence matching performance associated with high degrees of perceptual asynchrony and low degrees of complexity were underpinned by a partially different set of brain regions. In light of earlier studies, our results suggest that while regions in the DLPFC are modulated by differences in memory load due to stimulus asynchrony, areas traditionally thought to be involved in speech production and recognition, such as the inferior frontal and superior temporal cortex, are modulated by the complexity of the temporal signal properties. Our study also provides evidence for region-specific processing roles within the CCN. In future studies, it will be important to investigate brain activity during different levels of stimulus complexity to explore whether the relationship between stimulus complexity and brain activation is linear or more multifaceted. Furthermore, future studies should investigate patient populations with lesions in the identified brain areas to substantiate the present findings.

## Synthesis

Reviewing Editor: Christophe Bernard, INSERM & Institut de Neurosciences des Systèmes Decisions are customarily a result of the Reviewing Editor and the peer reviewers coming together and discussing their recommendations until a consensus is reached. When revisions are invited, a fact-based synthesis statement explaining their decision and outlining what is needed to prepare a revision will be listed below. The following reviewer(s) agreed to reveal their identity: Sophie Molholm, Ross Maddox.

Dear authors,

We all agree that you have addressed all issues.

There are a couple of minor issues easily addressed, regarding some sentences that could be dropped.

From the significance statement: “This study not only shows that temporal complexity and synchrony of audiovisual streams affects the formation of multisensory precepts” -- the formation of multisensory percepts is not measured here (and percepts is misspelled) and so this statement should be removed.

The relevance of this statement is not clear; the statement could just be dropped: “ Furthermore, coinciding audiovisual stimuli evoke larger BOLD responses in multisensory as well as primary sensory cortical areas than unisensory stimuli (Baumann & Greenlee, 2007).”

Line 72: “but would also help to further elucidate the functional neuroanatomy of multisensory perception.” Not really. Comparison with unisensory conditions would be necessary for this statement to be accurate.
